# Simultaneous development of COVID-19 pneumonia and pulmonary metastasis in a known case of chondrosarcoma: a case report

**DOI:** 10.1186/s13256-021-02753-1

**Published:** 2021-04-28

**Authors:** Afshin Rakhsha, Zahra Mahboubi-Fooladi, Anya Jafari

**Affiliations:** 1 Department of Radiation Oncology, Shahid Beheshti University of Medical Sciences, Shohada‐e Tajrish Hospital, Shahrdari St, 1989934148 Tehran, Iran; 2 Department of Radiology, Shahid Beheshti University of Medical Sciences, Tehran, Iran

**Keywords:** Case report, COVID-19, SARS-CoV-2, Chondrosarcoma, Pulmonary metastasis

## Abstract

**Background:**

The outbreak of coronavirus disease 2019 (COVID-19) started in December 2020, and is a global problem now. There are several sets of established data regarding computed tomography (CT) findings in COVID-19 pneumonia with many differential diagnoses. During the early days of the pandemic, there was little data regarding lung CT features of COVID-19 in a cancer patient. In this paper, we described a rare case of simultaneous presentation of COVID-19 with pulmonary metastasis.

**Case presentation:**

A Persian patient with a history of chondrosarcoma presented to our clinic during the COVID-19 pandemic with a new-onset cough. He had experienced no recurrence during previous follow-up visits. Chest CT scan revealed numerous bilateral small peripheral and perilymphatic pulmonary nodules, unilateral ground-glass patch, and nodular interlobular septal thickening. Biopsy of the pulmonary nodules established pulmonary metastasis of chondrosarcoma origin, and pharyngeal reverse transcription polymerase chain reaction (RT-PCR) was positive for COVID-19.

**Conclusion:**

Pulmonary metastasis should be considered as a differential diagnosis of COVID-19 features in cancer patients in the pandemic era.

## Background

Coronavirus disease 2019 (COVID-19), caused by the novel severe acute respiratory syndrome coronavirus 2 (SARS-CoV-2), first appeared in Wuhan, China, characterized by acute mild to severe respiratory symptoms. Immunocompromised patients are at high risk of COVID-19 infection. Differentiating COVID-19 lung features from lung involvement by metastasis is especially challenging for oncologists during the pandemic. Here we present a known case of sarcoma with challenging chest CT scan findings.

## Case presentation

A 54-year-old Iranian man, with a history of chondrosarcoma of the left lower limb, presented to our clinic with a chief complaint of cough in March 2020 during the outbreak of COVID-19. The patient had presented with a 6 cm mass in the left anterior thigh 3 years earlier, and at that time, after an incisional biopsy, he underwent wide local excision of the primary tumor. Pathology examination revealed extraskeletal myxoid chondrosarcoma with moderate differentiation, so he received postoperative radiotherapy, and as there was no evidence of recurrence, was followed up every 3 months until the outbreak. Familial history and other medical history were unremarkable.

He complained of progressive dry cough and sweating. No history of fever, weight loss, or any other symptoms was reported. Examination of vital signs revealed a respiratory rate of 20 breaths/minute, blood pressure of 100/60 mmHg, oral temperature of 36.9 °C, and pulse rate of 120 beats/minute. On lung auscultation, he had bilateral fine rales. Blood tests revealed leukopenia (3.2 × 10^9^/ L, normal: 4–9 × 10^9^/L) and lymphopenia (1.2 × 10^9^/L).

Based on two main differential diagnoses of pulmonary metastasis or SARS-CoV-2 infection, he underwent a chest computed tomography (CT) scan with intravenous contrast and SARS-CoV-2 reverse transcription polymerase chain reaction (RT-PCR).

**Fig. 1:**
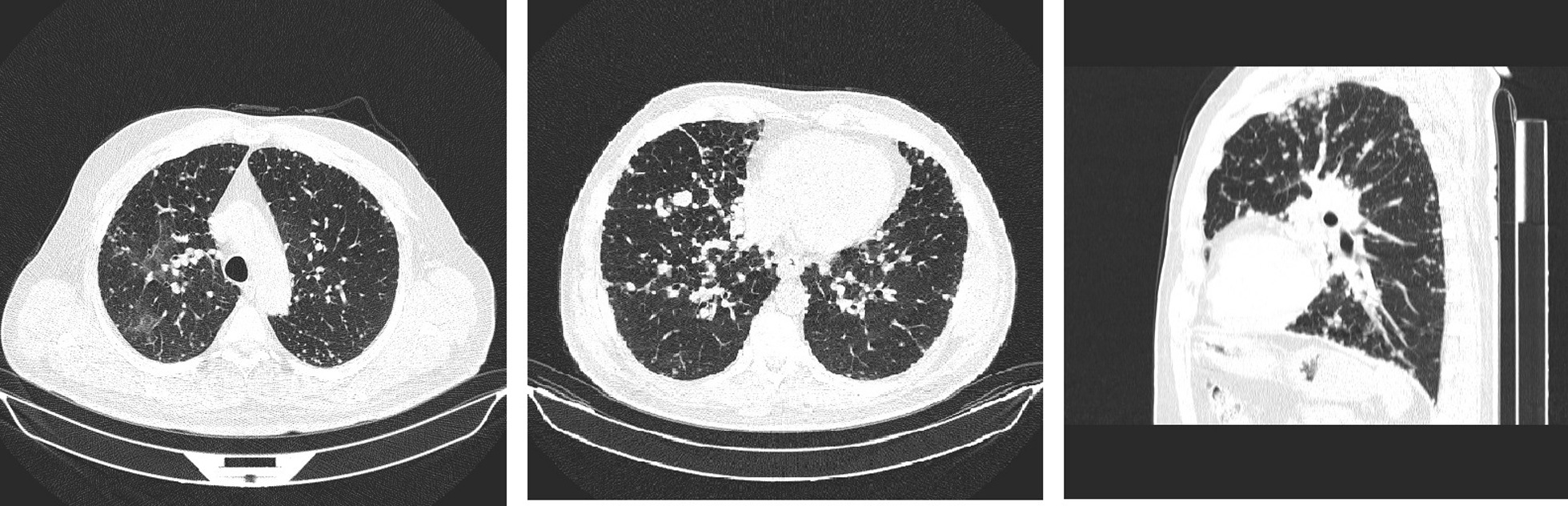
Axial and sagittal views of chest CT scan of a patient with COVID-19 and pulmonary metastasis show nodules and grand glass opacities

The SARS-CoV-2 RT-PCR test result, based on a pharyngeal swap sample, was positive. Chest CT revealed numerous bilateral small peripheral and perilymphatic pulmonary nodules, unilateral ground-glass patch, and nodular interlobular septal thickening. There was no evidence of mediastinal lymphadenopathy or pleural effusion (Fig. 1).

Due to the challenging CT findings, it was difficult to rule out pulmonary metastasis in this case, so several lung biopsies were taken, and the pathology report revealed a recurrence of myxoid sarcoma in the lung. The patient was cared for as a COVID-19-infected case, and subsequently palliative chemotherapy was administered.

## Discussion

Since SARS-CoV-2 infection was initially reported in China in January 2020, several clinical manifestations have been documented [1]. The most common presentations are fever (88.7%), dry cough (57.6%), and dyspnea [2]. It is expected that immunocompromised patients are at increased risk of infection in comparison with the general population [3, 4]. Despite reports about SARS-CoV-2 infection in cancer patients, it is not known whether the infection presents differently from that in the normal population [5], and the same is true about the CT features. In our case, the last cancer treatment was localized radiotherapy 3 years earlier, and the patient was monitored as cancer-free before the pandemic, so it was reasonable to consider COVID-19 infection as the differential diagnosis of dry cough in this patient.

COVID-19 pneumonia can have a wide spectrum of CT findings including ground-glass opacity (GGO) and rarely, discrete pulmonary nodules, as in our patient [6]. There are many publications on this issue, and some rare radiologic presentations have been described. The most common lung features of COVID-19 pneumonia are bilateral and multi-lobe involvement with subpleural GGO ± consolidations [7]. On the other hand, metastatic chondrosarcoma of bone can show bilateral lobulated nodules with a bilateral diffuse distribution pattern and internal calcification [8]. Unilateral one-lobe involvement with GGO is mostly in favor of cancer [9]. It is not clear whether the pulmonary involvement of COVID-19 in cancer patients is different from that in the general population. Therefore, the development of new pulmonary nodules in a patient with a history of malignancy during the pandemic raises the need to evaluate each of these diagnoses.

Due to our challenge in the differentiation of these two entities on CT scan, nonspecific clinical symptoms, and reported false-positive COVID-19 RT-PCR results, we decided to perform lung biopsy, which established the metastasis. The patient received azithromycin and tocilizumab for COVID-19 infection, and after recovery, palliative chemotherapy was planned.

## Conclusion

Although cancer patients are more likely to be infected with COVID-19, pulmonary metastasis should be kept in mind as a differential diagnosis of COVID-19 lung features, especially with pulmonary nodules in these patients during the pandemic.

## Data Availability

Data sharing is not applicable to this article, as no data sets were generated or analyzed during the current study.

## Abbreviations

COVID-19: Coronavirus disease 2019
CT scan: Computed tomography scan
RT-PCR: Reverse transcription polymerase chain reaction
GGO: Ground-glass opacity
